# MRI-Based Radiomics Features to Predict Treatment Response to Neoadjuvant Chemotherapy in Locally Advanced Rectal Cancer: A Single Center, Prospective Study

**DOI:** 10.3389/fonc.2022.801743

**Published:** 2022-05-12

**Authors:** Bi-Yun Chen, Hui Xie, Yuan Li, Xin-Hua Jiang, Lang Xiong, Xiao-Feng Tang, Xiao-Feng Lin, Li Li, Pei-Qiang Cai

**Affiliations:** ^1^Department of Medical Imaging, Collaborative Innovation Center for Cancer Medicine, State Key Laboratory of Oncology in South China, Sun Yat-sen University Cancer Center, Guangzhou, China; ^2^Department of Colorectal, Collaborative Innovation Center for Cancer Medicine, State Key Laboratory of Oncology in South China, Sun Yat-sen University Cancer Center, Guangzhou, China; ^3^Department of Ultrasound, Collaborative Innovation Center for Cancer Medicine, State Key Laboratory of Oncology in South China, Sun Yat-sen University Cancer Center, Guangzhou, China

**Keywords:** rectal cancer, treatment response, neoadjuvant chemotherapy, nomogram, magnetic resonance imaging radiomics

## Abstract

This is a prospective, single center study aimed to evaluate the predictive power of peritumor and intratumor radiomics features assessed using T2 weight image (T2WI) of baseline magnetic resonance imaging (MRI) in evaluating pathological good response to NAC in patients with LARC (including Tany N+ or T3/4a Nany but not T4b). In total, 137 patients with LARC received NAC between April 2014 and August 2020. All patients were undergoing contrast-enhanced MRI and 129 patients contained small field of view (sFOV) sequence which were performed prior to treatment. The tumor regression grade standard was based on pathological response. The training and validation sets (n=91 vs. n=46) were established by random allocation of the patients. Receiver operating characteristic curve (ROC) analysis was applied to estimate the performance of different models based on clinical characteristics and radiomics features obtained from MRI, including peritumor and intratumor features, in predicting treatment response; these effects were calculated using the area under the curve (AUC). The performance and agreement of the nomogram were estimated using calibration plots. In total, 24 patients (17.52%) achieved a complete or near-complete response. For the individual radiomics model in the validation set, the performance of peritumor radiomics model in predicting treatment response yield an AUC of 0.838, while that of intratumor radiomics model is 0.805, which show no statically significant difference between then(P>0.05). The traditional and selective clinical features model shows a poor predictive ability in treatment response (AUC=0.596 and 0.521) in validation set. The AUC of combined radiomics model was improved compared to that of the individual radiomics models in the validation sets (AUC=0.844). The combined clinic-radiomics model yield the highest AUC (0.871) in the validation set, although it did not improve the performance of the radiomics model for predicting treatment response statically (P>0.05). Good agreement and discrimination were observed in the nomogram predictions. Both peritumor and intratumor radiomics features performed similarly in predicting a good response to NAC in patients with LARC. The clinic-radiomics model showed the best performance in predicting treatment response.

## Introduction

Rectal cancer is one of the most common malignant neoplasms and the second leading cancer-related cause of death worldwide (1). Locally advanced rectal cancer (LARC) accounts for approximately 70% of newly diagnosed rectal cancer cases annually, which is defined as T3-4Nany or TanyN+, regardless the status of the CRM (2). Following neoadjuvant fluorouracil-based chemoradiotherapy (CRT), total mesorectal excision (TME) and adjuvant chemotherapy are the recommended standard treatments for LARC before. However, several large prospective trials, including RAPIDO (3), PRODIGE 23 (4) have brought neoadjuvant chemotherapy to the fore as a new standard to LARC. Furthermore, recent results indicated that, compared to nCRT, neoadjuvant chemotherapy (NAC) showed no statistically significant difference in terms of 3-year local recurrence (8.3% vs 7.0%~ 8.0%), disease-free survival (DFS) (73.5% vs 72.9%~77.2%), and overall survival (OS) (90.7% vs 89.1%~91.3%) between the three arms (5). Even Habr-Gama and colleagues suggest the ‘wait and watch’ strategy for patients with LARC with a clinical complete response (cCR) after neoadjuvant chemoradiotherapy (nCRT) (5). Although the rate of pathological complete response (pCR) in the CRT group was higher than that in the NAC group, higher toxicity and more postoperative complications were observed in patients who received only radiotherapy (6, 7). Predicting patients who could achieved CR undervent NAC before operation is of great clinically meaning. It may indicates that the specific patients could avoid the unnecessary radiotherapy (8).

However, pCR (pathology complete response) can only be confirmed in the resected specimens after surgery.Magnetic resonance imaging (MRI) has merged as a dominant method of pelvic imaging in rectal cancer for its superb soft tissue contrast between tumor and other soft tissue (9). Besides, MRI is particular accurate in assessing the distance between the tumor and the mesorectal fascia with sensitivity and specificity up to 94% and 76% respectively (10). Furthermore, 3.0T MRI scanner perform better than the 1.5T MRI in the visual assessment of the complete response patients of rectal cancer (10). In spatially and temporally heterogeneous solid cancers, invasive biopsies specimens cannot reflect the overall characteristics of the tumor, which limits the use of invasive biopsy based molecular assays but gives huge potential for medical imaging (11). Radiomics, as a new term, was first proposed by Lambin et al. in 2012 (12). Radiomics can convert traditional radiological images into data that can be further analysis. The workflow includes multi-steps: images acquisition, image segmentation, features extraction and selection, model construction and validation. The aim of radiomics is to translate medical images into quantitative data, which may reveal a deeper information of the tumor (13). With its ability to perform high-throughput extraction of image features derived from radiographic images, radiomics can provide a non-invasive method of describing intra-tumoral heterogeneity (12). Previous studies (14–18) have estimated the predictive performance of magnetic resonance imaging (MRI) or computed tomography (CT) features to evaluate tumor treatment response. In these studies, most examinations were focused on LARC after nCRT, which is of little significance for making decisions regarding NAC. In addition, previous radiomics studies of LARC focused on the intertumoral region alone, and information regarding peritumoral radiomics features was overlooked. Recently, in other cancers, the peritumoral area has been used to predict the treatment response. For example, Khorram et al. (19) used combined peri- and intratumoral radiomics models to predict the response to chemotherapy in lung adenocarcinoma and indicated that radiomic features extracted within the nodule and border (from the baseline CT scan) performed well in predicting treatment response in association with time to progress (TTP) and overall survival (OS). Hu et al. (20) showed that, in patients with esophageal squamous cell carcinoma, a model combining intra- and peritumoral radiomics showed good performance in predicting pCR following NAC (0.852 (95% CI, 0.753-0.951). At present, to the best of our knowledge, only a few studies have focused on peritumor radiomics research, and its role in predicting treatment response to NAC has not been definitively demonstrated. Therefore, we aimed to establish the model based on radiomics of the intratumor and peritumor to predict the efficacy of NAC in LARC. Prediction of good response to NAC can reduce radiotherapy-related toxicity and the economic burden of the patients. Our findings, if confirmed, may help identify good response patients and lower the overtreatment rate.

## Materials and Methods

### Patient Population

**Figure 1:**
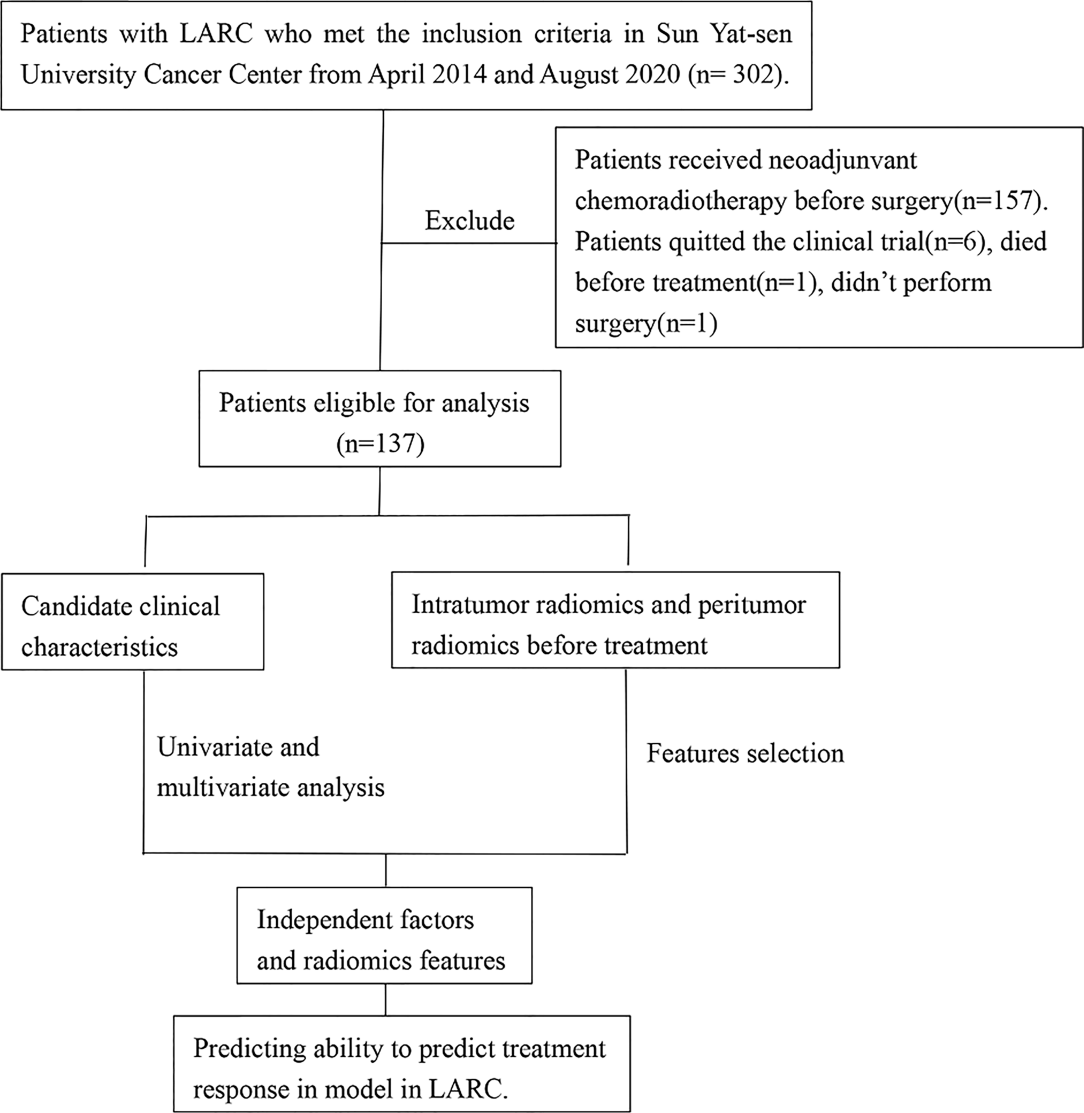
Flow chart of the study.

The study was approved by the Ethical Committee of the Sun Yat-sen University Cancer Center. This was a secondary analysis based on prospective research data (Approval no. 5010-2014-013). The ethical principles of the Declaration of Helsinki were followed in conducting work involving human participants enrolled in this study. Informed consent was obtained from each patient prior to treatment and participation. The study cohort was enrolled between April 2014 and August 2020. The patients were recruited based on the following inclusion criteria: (1) pathologically confirmed single primary rectal cancer; (2) clinical diagnosis of LARC (defined as the tumor invading the muscularized layer of the intestinal wall with positive peripheral lymph node metastasis (T2 N+) or primary tumor invading the subserosa regardless of the status of the lymph node (T3-4aNany); (3) no distant metastasis; (4) initial pretreatment MRI of the pelvis; (5) no other malignant cancers; (6) no anti-cancer treatment in other clinical centers; (7) an Eastern Cooperative Oncology Group score of 0–1; (8) age between 18 and 75 years. The exclusion criteria were as follows: (1) preoperative staging evaluating whether the tumor had invaded the surrounding tissues or organs (T4b); (2) severe hypertension with poor control; (3) history of viral infection, including human immunodeficiency virus or chronic hepatitis B or C; (4) arrhythmia requiring antiarrhythmic treatment (except *via* blockers or digoxin), myocardial ischemia (i.e., myocardial infarction in the last 6 months), or symptomatic coronary artery disease with heart failure exceeding New York Heart Association level II criteria; and (5) a history of pelvic or abdominal radiotherapy to exclude the influence of other serious diseases and treatment history on the treatment outcomes. The patient selection process for this study is summarized in **Figure 1**. A total of 302 eligible patients were recruited from April 2014 and August 2020. Of these, a further 165 patients were ineligible and were excluded, including 157 patients received CRT, 6 patients quitted the clinical trial, 1 patient died before treatment and 1 patient didn’t perform surgery, and finally data collection could be complete on a total 137 participants (83 men and 54 women).

The following variables were obtained from patient medical charts: age, sex, body mass index (BMI), history of smoking, initial carcinoembryonic antigen (CEA) and carbohydrate antigen 19-9 (CA19-9) levels (as determined by MRI), the date and cycle of chemotherapy, surgery date, and tumor regression grade (TRG). Blood samples were collected from all patients within one week of treatment. In addition, the date of the baseline MRI, clinical T and N staging, the distance from the tumor to the anal edge, the long and short diameter of the tumor, the status of mesorectal fascia (MRF) invasion, and extramural vascular invasion as determined by MRI were obtained.

### Neoadjuvant Chemotherapy and Pathological Assessment

The chemotherapy regimen implemented within the current study was the CapeOx plan (oxaliplatin 30 mg/m^2^, day 1; capecitabine 850–1,000 mg/m^2^, bid, days 1–14). There were breaks of three weeks between cycles. The total number of cycles ranged between two and four. Upon completion of chemotherapy, TME surgery was performed.

Two experienced gastrointestinal cancer pathologists (Dr. Xi and Dr. Wu) with 8 and 12 years of diagnosis experience, respectively, reviewed and evaluated all resected specimens. Clinical and MRI data were also evaluated by these pathologists. TRG was evaluated based on the TRG system proposed by Mandard et al. (21) TRG was quantitated in five grades: TRG1 (complete regression) showed absence of residual cancer and fibrosis extending through the different layers of the bowel wall; TRG2 was characterized by the presence of rare residual cancer cell scattered through the fibrosis; TRG3 was characterized by an increase in the number of residual cancer cells, but fibrosis still predominated; TRG4 showed residual cancer outgrowing fibrosis; and TRG 5 was characterized by absence of regressive changes. Considering that a good response to NAC can lead to avoiding radiotherapy and that poor responders may need further treatment or assess other treatment options, patients were divided into two response groups: good responders (TRG 1–2 disease, no or rare tumor cells remaining) and poor responders (TRG 3–5 disease, moderate to extensive residual cancer cells). A consensus was reached in cases involving uncertainties in evaluating the pathology specimens.

### MRI and Image Evaluation

Patients underwent rectal MRI prior to NAC. The time from the MRI to the start of therapy was less than 2 weeks. Response: thank you very much for your question. All the MRI examinations were performed without bowel preparation, endorectal gel or spasmolytic drugs. Pretreatment MRI was performed using a 3.0 T MR scanner (Trio Tim, Siemens Healthcare, Malvern, PA, USA; Achicva 781-278, Philips, Cambridge, MA, USA) using two elements of the body matrix coil as well as two elements of the spine matrix coil, or was performed using a 3.0 T system (Discovery 750, 750 W, SIGNA Pioneer GE Healthcare, Chicago, IL, USA; uMR 780, United Imaging, Shanghai, China) equipped with an eight-channel phased-array body coil in the supine position. A conventional rectal MRI protocol including diffusion-weighted imaging (DWI) and axial, coronal, and sagittal T2W images was implemented in all patients. Contrast-enhanced sequences were obtained. Detailed MRI protocol were list in **Table 1**.

**Table 1:** Detailed MRI protol.

MRI protocol			
Sequences	FOV(cm)	Slice gap	Slice sapcing
Axis T2WI without fat suppress, small FOV, thin layer, The upper bound included the entire sacral promontory, and the lower bound included the entire anus, area larger than 320*256	20	3	0.5
Axis T1WI without fat suppress, small FOV, thin layer, Turbo spin echo (TSE)	30-40	5	1
Axis DWI with fat suppress, b=800	30-40	5	1
Axis contrast-enhanced LAVA sequences with fat suppress	30-40	4	-2 ov

The features of tumor location, MRF, and extramural venous invasion (EMVI) were evaluated by two radiologists (Dr. Cai and Dr. Li with 12 and 16 years of experience respectively in rectal cancer imaging), and the double-blind principle was applied. The rectum extends from the anal verge (AV) to a distance 15 cm cranially and can be divided into upper rectum (10.1-15cm from AV), mid rectum (5.1-10cm from AV) and lower rectum (0 to 5 cm from AV). The MRF positive was defined that the distance was less than 1mm from the tumor to mesorectal fascia. The extramural venous invasion was assess based on the EMVI scoring system raised by Smith et al. (22). Score 0-2 was defined EMVI negative and score 3-4 was defined EMVI positive.

### Tumor Segmentation

All regions of interest (ROIs) were manually evaluated *via* the T2 Weighted image (T2WI) in each slice of the MRI for 3D segmentation. The ROI 1 is the segmentation for the whole tumor and ROI 2 was the segmentation for the tumor bed, which was defined the area that the tumor invaded to the mesorectal. Image segmentation was performed using the open-source software ITK-SNAP (version 3.8.0, www.itksnap.org/; developed at the University of Pennsylvania and the University of North Carolina at Chapel Hill). Digital imaging and communications in medicine images were obtained prior to treatment. Small field of view, high-resolution axial T2W sequence is a priority for segmentation. If this sequence could not be obtained, the T2W sequence best displaying the tumor was used for segmentation (including coronal, sagittal, or large field images of the non-high-resolution axial).

### Radiomics Feature Extraction

**Figure 2:**
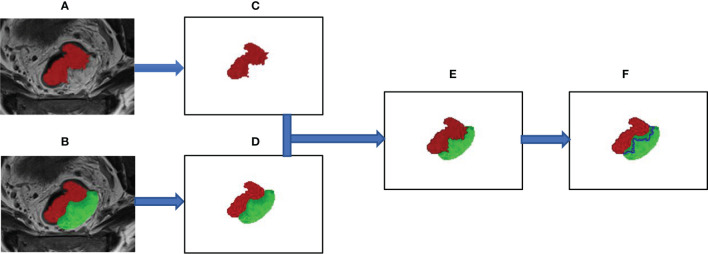
The segmentation process of the region of interest for intratumor and peritumor. **(A)** The whole tumor was manually segmented on axial T2-weighted images and labeled as “intratumor” area (the red area). **(B)** Manually outline the area of the tumor bed (the green area), which is defined as the mesorectal area that the tumor has invaded. **(C)** Show the outline of the entire tumor. **(D–F)** The edge link to the “tumor bed” of “tumor” was dilated by 5 mm and subtracted to obtain the “peritumor” tissues (the blue area).

ROI segmentation was used for radiomics feature extraction. Radiomics feature extraction was performed using the “PyRadiomics” package in Python (version 3.0, Wilmington, DE, USA; https://pyradiomics.readthedoc s.io/). Features extracted from the volume of the entire tumor were defined as intertumoral features. Based on the whole tumor, peritumor features were acquired by expanding 5 mm from the border of the tumor to the tumor bed. The process of intratumor and peritumor ROI segmentation is shown in **Figure 2**.

To avoid data heterogeneity bias, all MRI data were subjected to imaging normalization (the intensity of the image was scaled to 0–100) and resampled to the same resolution (1mm×1mm×1 mm) before feature extraction. For each ROI, six feature classes [251 first order statistics, and texture classes (including 336 gray level cooccurrence matrix, 224 gray level run length matrix, 224 gray level size zone matrix, 196 gray level dependence matrix, and 70 neighboring gray tone difference matrix)] were calculated, which resulted in a total of 1301 radiomics features for each scan. The file for the extraction process is included in the **Supplementary Material**.

### Feature Selection

**Figure 3:**
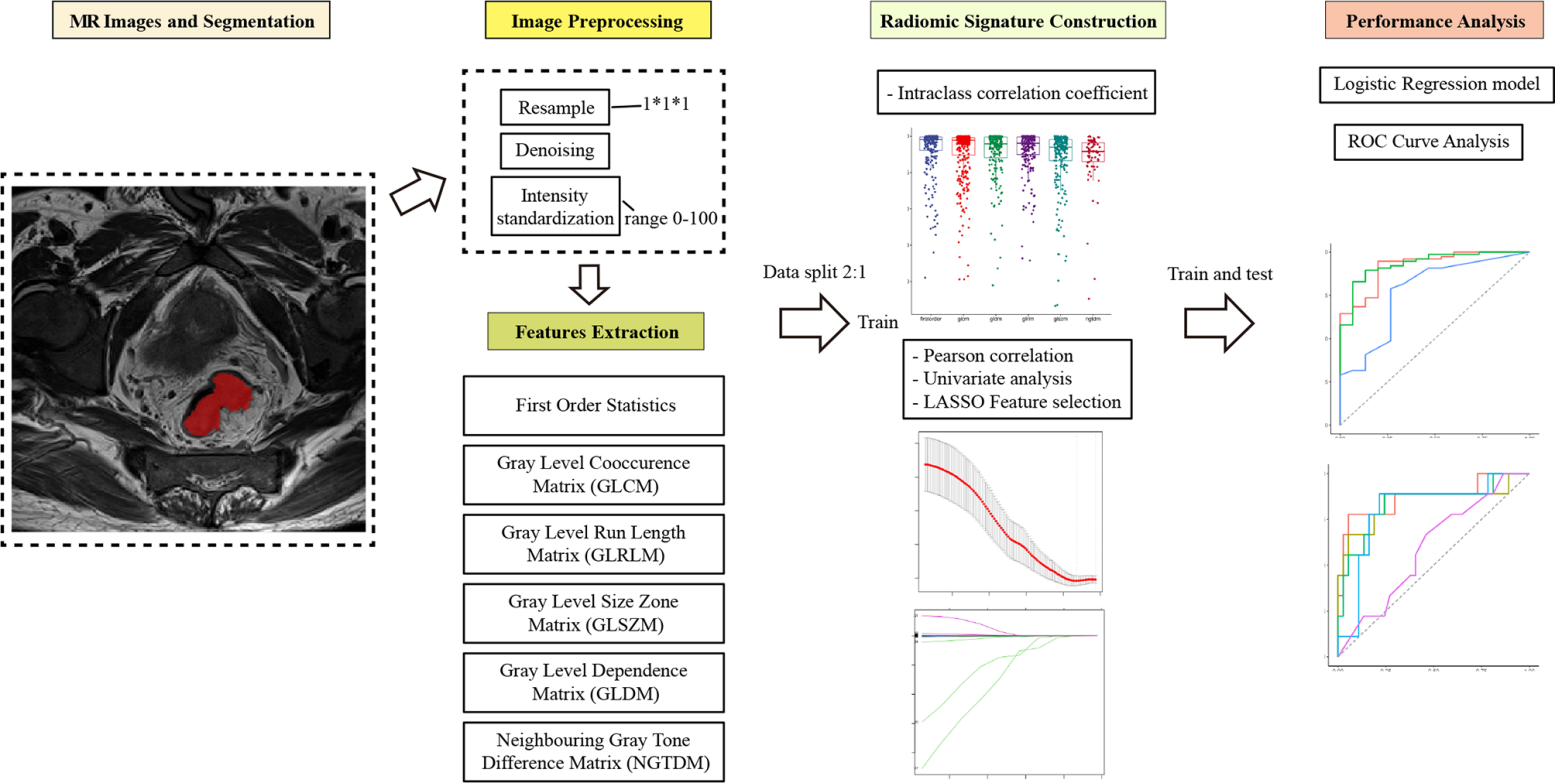
The workflow of the radiomics model construction.

The selection process was as follows to screen valuable features and reduce redundant, irrelevant features. First, it was necessary to perform standard scaling of the extracted features. Second, the inter-reader agreement was estimated using the interclass coefficient (ICC) between features extracted from the two segmentations. Only features with an ICC >0.70 were selected for the feature selection process. Third, after conducting Pearson correlation, univariate analysis, and the application of the least absolute shrinkage and selection operator (LASSO) algorithm, the most useful predictive parameters were selected to construct the delta-radiomics signature. Finally, multivariate logistic regression was used to generate the prediction model. The workflow of the radiomics model construction was showed in **Figure 3**.

### Statistical Analysis

X^2^ or Fisher’s exact tests were used for comparing categorical variables, while the Kruskal–Wallis test was used to compare numeric variables. A receiver operating characteristic curve (ROC) analysis was performed to estimate the predictive performance of different models, which was calculated as the area under the curve (AUC). Calibration curve were evaluated using calibration and decision curves. Univariate and multivariate logistic analyses were performed to clarify the relationship between clinical parameters and pCR. Statistical significance was defined as a two-tailed p-value of <0.05. Python (version 3.7) and R statistical software (version 3.3.3; Vienna, Austria) were used for graphical depiction and statistical analysis, respectively.

## Results

### Clinical Characteristics

The clinical characteristics of the enrolled patients are summarized in **Table 2**. In total, 137 patients were recruited in this study. Participants were randomly divided into training (n=91) and validation sets (n=46). The mean age of the patients was 57 (range: 30–77) years; 83 (60.1%) were men, and 55 (39.8%) were women. There was no statistically significant difference in the good response rate between the training and validation cohorts (16.5% vs. 19.5%, p=0.834). Except for maximal lymphnode (max LN), statistically significant differences were not observed in all clinical features when comparing the training and validation cohorts. mulivariate factor analysis revealed that the CEA level, T stage, and tumor invasion circumference (TIC) were relative to TRG (p<0.05). (**Table 3**).

**Table 2:** Clinical characteristics of patients in the training and validation set.

Characteristics	Training	Validation	*P*
	(N = 91)	(N = 46)	
Gender			0.54
Male	53	30	
Female	38	16	
Age			0.90
<60	49	26	
≥60	42	20	
BMI			0.64
<18.5	8	6	
18.5-24	56	25	
>24	27	15	
Smoking			0.28
no	74	33	
yes	17	13	
Family history			0.85
no	72	35	
yes	19	11	
HB (g/l)			0.78
<120	15	6	
≥120	76	40	
CEA (ng/ml)			0.99
<5	56	28	
≥5	35	18	
CA19-9 (u/ml)			0.53
<35	82	39	
≥35	9	7	
T stage			0.42
T3a	28	13	
T3b	24	16	
T3c	4	0	
T4a	35	17	
Distance (cm)			0.55
≤5	14	8	
5.1-10	65	29	
>10	12	9	
TIC			0.69
1/4	1	1	
2/4	26	11	
3/4	41	25	
4/4	23	9	
MRF			0.64
negative	77	32	
positive	14	14	
EMVI			0.45
negative	72	32	
positive	19	14	
LN metastasis			0.20
negative	63	26	
positive	28	20	
max LN (mm)			0.04*
<5	48	15	
≥5	43	31	
TRG			0.83
0	76	37	
1	15	9	

*p < 0.05.

**Table 3:** Univariate and multivariate analyses of the clinical characteristics.

Characteristics	univariate			Multivariate		
	HR	CI 95%	P	HR	CI 95%	P
Gender						
Male						
Female	1.27	0.41-3.90	0.67			
Age						
<60						
≥60	1.03	0.33-3.13	0.97			
BMI						
<18.5						
18.5-24	1.34	0.20-26.63	0.80			
>24	1.59	0.21-33.19	0.69			
Smoking						
Yes						
No	0.63	0.09-2.60	0.56			
Family history						
yes						
no	2.21	0.61-7.34	0.20			
HB (g/l)						
<120						
≥120	0.75	0.20-3.64	0.69			
CEA (ng/ml)						
<5						
≥5	0.20	0.03-0.79	0.04*	0.23	0.03-1.05	0.08**
CA19-9(u/ml)						
<35						
≥35	0.28	0.02-1.52	0.23			
T stage						
T3a						
T3b	0.19	0.03-0.86	0.05*	0.23	0.03-1.15	0.10
T3c	0.70	0.03-6.42	0.77	0.83	0.03-12.02	0.89
T4a	0.20	0.04-0.76	0.03*	0.25	0.05-1.09	0.08**
LN metastasis						
positive						
negative	1.15	0.33-3.64	0.81			
Distance (cm)						
≤5						
5.1-10	1.22	0.28-8.56	0.81			
>15	1.20	0.13-11.54	0.87			
TIC						
≤2/4						
3/4	0.22	0.05-0.76	0.02*	0.26	0.06-1.02	0.06**
4/4	0.19	0.03-0.86	0.05*	0.53	0.06-3.39	0.52
MRF						
positive						
nagative	1.48	0.30-5.62	0.59			
MRF invasion						
Tumor						
LN	0.70	0.15-2.57	0.61			
tumor deposit	1.23	0.06-9.45	0.86			
other	2.45	0.11-28.1	0.48			
EMVI						
positive						
negative	0.94	0.20-3.40	0.93			
max LN (mm)						
<5						
≥5	0.97	0.31-2.97	0.96			

*p < 0.05; **p < 0.1.

### Feature Selection and Radiomics Signature Construction

In total, 1,301 radiomics features were retrieved from intratumor and peritumor images conducted *via* T2WI. We performed Pearson correlation and univariate analyses to screen the correlative features and eliminate features with low reproducibility. LASSO analysis was then used to select the features from among the screened features (**Supplementary Figure 1**). Finally, following backward elimination, 9 and 10 features were selected from the peritumor and intratumor images, respectively (**Table 4**). The remaining features are listed in **Supplementary Table 1**.

**Table 4:** Numbers of features that remained after each selection step for radiomics signature construction.

Feature selection steps	Peritumor Features	Intratumor Features
Before selection	1301	1301
ICC	1275	1143
Pearson correlation	345	336
Univariate analysis	56	37
LASSO	14	16
Backward elimination	9	10

ICC, interclass correlation coefficient; LASSO, least absolute shrinkage and selection operator.

Eventually, the traditional clinical characteristics, selective clinical characteristics and radiomics signature were used to constructed the predictive model, including the traditional clinical model (including T and N stage), selective clinical model (including T stage, CEA level, TIC), intratumor radiomics model, peritumor radiomics model, combined radiomics model (including intra-peritumor radiomics) and clinic-radiomics model (selective clinical characteristics combined intra-peritumor radiomics).

### Models Performance

The traditional clinical model yields an AUC of 0.677 (95% confidence interval [CI] 0.527–0.827) in training set and an AUC of 0.701 (95% CI 0.565-0.837) in validation set. When considering the selective clinical model, the results suggest that the AUC in the training set is 0.775 (95% CI 0.637-0.913) and that is 0.596 (95% CI 0.637-0.913) in validation set. In the training cohort, intratumor radiomics features model yielded an AUC of 0.932, whereas that of the peritumor model was 0.921. Furthermore, the two models yielded an AUC of 0.805 and 0.838, respectively, in the validation set (**Table 5**).

**Table 5:** The AUC value of clinical characteristics and radiomics model.

variable	Training			
	AUC	95% CI	PI	P2
R1	0.921	0.852-0.990	reference	0.751
R2	0.932	0.870-0.995	0.751	reference
Clinis	0.775	0.637-0.913	0.066	0.044
T+N	0.677	0.527-0.827	< 0.001*	< 0.001*
**variable**	**Validation**			
	**AUC**	**95% CI**	**PI**	**P2**
R1	0.838	0.661-1.000	reference	0.583
R2	0.805	0.633-0.976	0.583	reference
clinics	0.596	0.396-0.796	0.079	0.125
T+N	0.521	0.279-0.763	0.024*	0.047*

R1, stand for peritumor radiomics; R2, stand for intratumor radiomics. T, T stage; N, N stage; CEA, carcinoembryonic antigen; Clinics, combined the selective clinical characterisitics, including CEA、Tstage and TIC.

*P < 0.05.

Compared to selective clinical model and the intratumor radiomics model individually, the combined selective clinic-intratumor radiomics model achieved the highest AUC (0.940, 95% CI 0.882–0.997) in the training set, whereas the AUC value is 0.781(95% CI 0.603–0.959) in the validation set. Besides, The AUC of the selective clinic-peritumor radiomics model for training set is 0.932 (95% CI 0.871–0.992) and that of the validation set is 0.844 (0.667-1.000), which is statistically significantly higher than that of the selective clinical model (p=0.025) (**Table 6**).

**Table 6:** The AUC of selective clinical model compared to radiomics model.

variable	Training			Validation		
	AUC	95%CI	P	AUC	95%CI	P
R1+clinics	0.932	0.871-0.992	reference	0.844	0.667-1.000	reference
R1	0.921	0.852-0.990	0.400	0.838	0.661-1.000	0.781
clinics	0.775	0.637-0.913	0.040	0.596	0.396-0.796	0.025*
R2+clinics	0.940	0.882-0.997	reference	0.781	0.603-0.959	reference
R2	0.932	0.870-0.995	0.392	0.805	0.633-0.976	0.360
clinics	0.775	0.637-0.913	0.030	0.775	0.637-0.913	0.180

*p < 0.05.

The AUC of the combined radiomics model was 0.949 (95% CI 0.887–0.998) in the training cohort and 0.844 (95% CI 0.650–1) in the validation cohort, which was higher than that of the individual radiomics model. Compared to the combined radiomics model, the combined clinic-radiomics model, improved the AUC from 0.844 to 0.871 in the validation set (**Table 7**).

**Table 7:** The AUC of radiomics model and clinics model.

variable	Training			Validation		
	AUC	95%CI	P	AUC	95%CI	P
R1+R2+clinics	0.961	0.922-0.999		0.871	0.706-1.000	
R1+R2	0.949	0.887-0.998	0.547	0.844	0.650-1.000	0.353
R1	0.921	0.852-0.990	0.322	0.838	0.661-1.000	0.789
R2	0.932	0.870-0.995	0.445	0.805	0.633-0.977	0.588
clinics	0.775	0.637-0.913	0.010*	0.596	0.396-0.796	0.001*

*p < 0.05.

**Figure 4:**
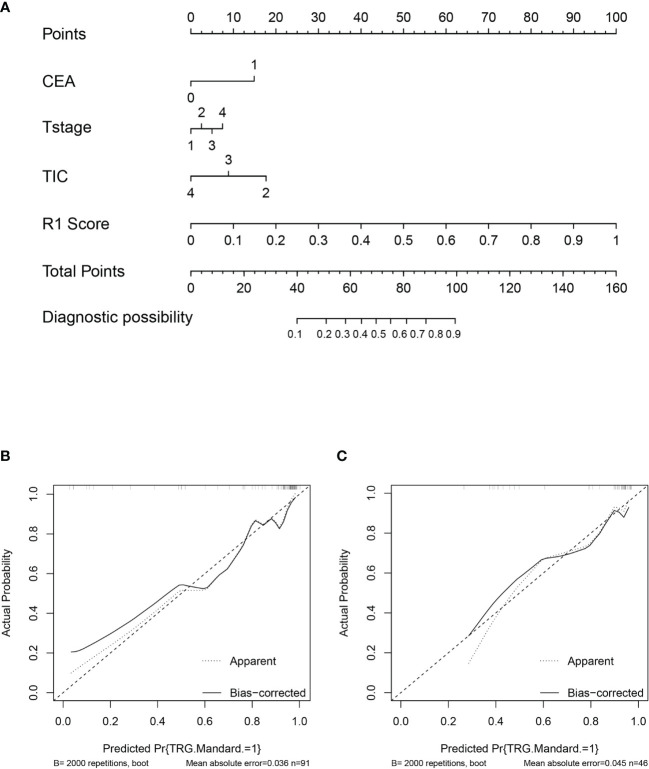
Nomogram based on the clinical characteristics and peritumor radiomics features in the prediction of response to neoadjuvant chemotherapy in locally advanced rectal cancer (LARC). **(A)** Nomogram based on peritumor radiomics clinical features. **(B)** The calibration curve for peritumor radiomics and clinical features in predicting treatment response for LARC in the training set. **(C)** The calibration curve for peritumor radiomics and clinical features in predicting treatment response for LARC in the validation set.

**Figure 5:**
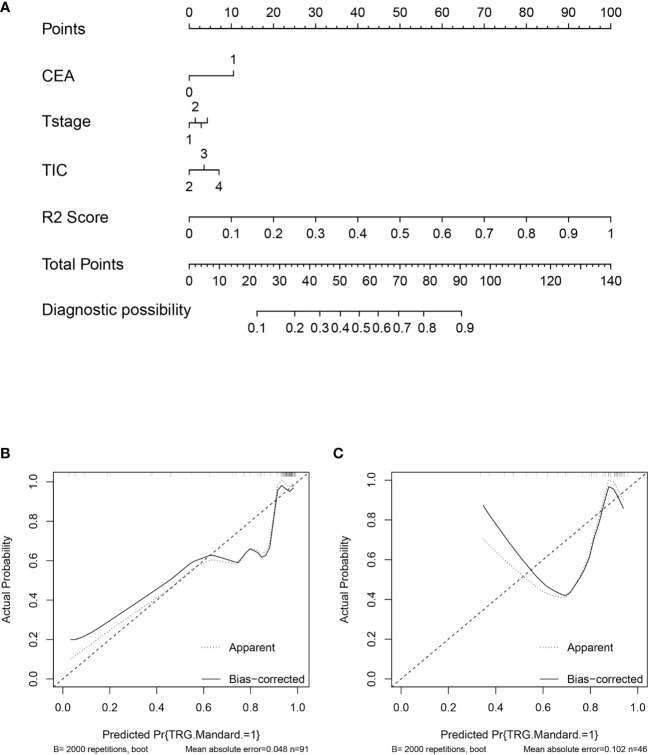
Nomogram based on the clinical characteristics and intratumor radiomics features in the prediction of response to neoadjuvant chemotherapy in locally advanced rectal cancer (LARC). **(A)** Nomogram based on clinical features and intratumor radiomics. **(B)** The calibration curve for intratumor radiomics and clinical features in predicting treatment response for LARC in the training set. **(C)** The calibration curve for intratumor radiomics and clinical features in predicting treatment response for LARC in the validation set.

**Figure 6:**
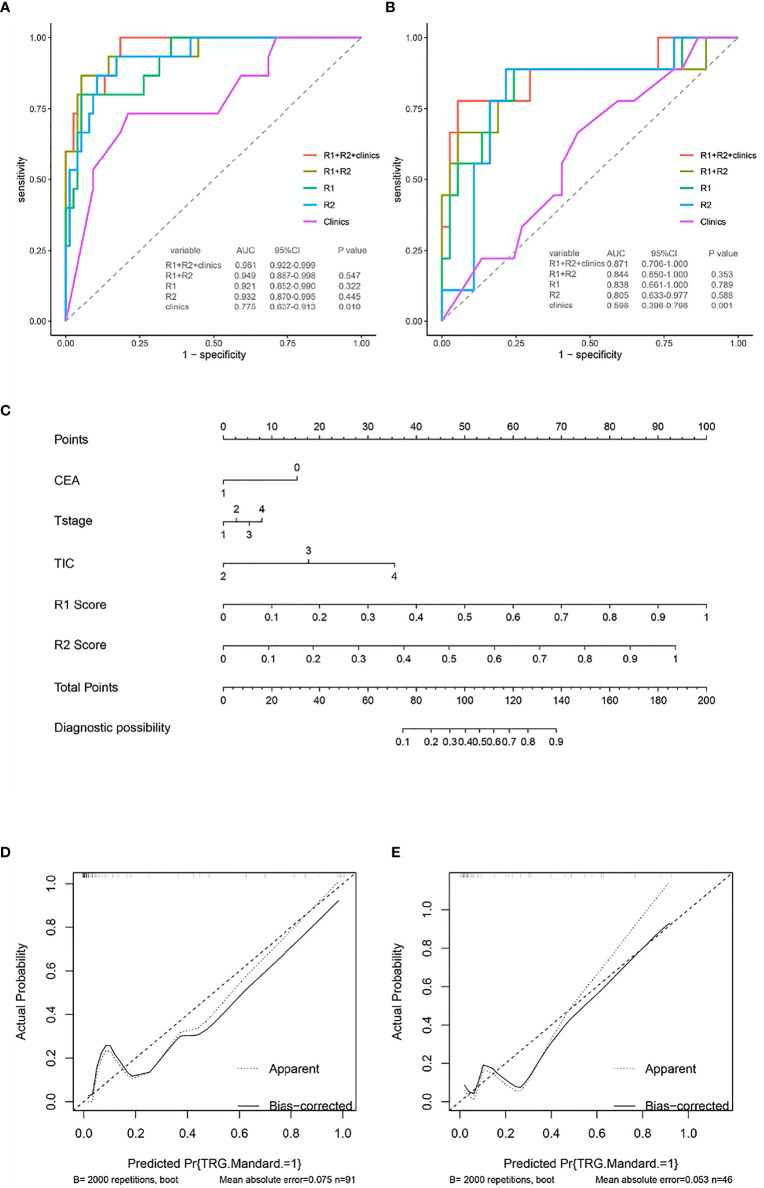
ROC and nomogram based on the clinical characteristics and intra-peritumor radiomics features in the prediction of response to neoadjuvant chemotherapy in locally advanced rectal cancer (LARC). **(A)** The receiver operating characteristic curve (ROC) for different models in predicting treatment response for LARC in the training set.**(B)** The ROC for different models in predicting treatment response for LARC in the validation set. **(C)** Nomogram based on the clinical characteristics and combined-radiomics features in the prediction of response to neoadjuvant chemotherapy in locally advanced rectal cancer (LARC). **(D, E)** The calibration curve for the models in predicting treatment response for LARC in the training set and validation set.

The nomogram of clinics-intratumor and clinics-peritumor for predicting good response as well as the calibration curve are presented in **Figures 4**, **5** respectively. The nomogram showed good performance in predicting the response to NAC. Good discrimination and good calibration for the probability of TRG were observed in the validation set with respect to clinics-peritumor radiomics, with an AUC of 0.838. However, in the validation set, our nomogram for the clinics-intratumor models did not achieve better discriminatory efficiency than that of the peritumor models. The nomogram of the clinic-radiomics model showed good discrimination in predicting treatment response in the validation set as well (**Figures 6 A–E**).

## Discussion

Pretreatment prediction of good treatment response is of great significance in pre-therapeutic decision-making. These models demonstrated that the performance of the radiomics signature model in predicting treatment response was far superior to that of the traditional or selective clinical features model. In addition, we found that peritumor radiomics model performed as well as intratumor radiomics model in predicting good treatment response after NAC. The combined clinic-radiomics model showed superior performance in predicting treatment response.

MRI is the image modality of choice when dealing with the primary staging and restaging after treatment in LARC. Although high-resolution MRI is recommended for T and N staging in patients with rectal cancer, the accuracy of staging is still unsatisfactory (18). The main challenge of MRI in assessment of pretreatment T stage is that differentiating early RC (T1-T2) from LARC (12). The study of Detering et al. showed that the accuracy of T stage of early RC evaluated by MRI is poor, with a 54% agreement to pathology (23). For the total T category, the sensitivity, specificity and accuracy of MRI assessment are 87%, 75% and 85% (24). In the assessment of N staging, a poor accuracy of 69% was observed in Detering’s study. Radiomics, which were obtained from the primary image of tumor, reflect number of characteristics, not only the shape and location, but also the tumor heterogeneity. Due to the lower accuracy of the staging of RC, it may lead to the poor performance in predicting the prognosis or the treatment response in rectal cancer. This hypothesis is consisted with our findings. Our study revealed that radiomics model has higher predictive performance than clinical model, no matter the traditional or the selective model, suggesting that radiomics could be a practical image biomarker for patients with LARC in predicting treatment response. However, lack of the standard protocol for MRI acquisition and uncertainties in tumor segmentation adversely limit the clinical application of radiomics.

Previous studies have identified that pretreatment T2W images play an important role in predicting treatment response (15, 16) in patients with LARC. However, these studies focused on patients with LARC who received nCRT. The rate of pCR to nCRT was higher than that to NAC in patients with LARC (6). In consideration of individual therapy strategies, screening patients who are likely to achieve a good response to NAC is important to avoid over-treatment. Moreover, most previous studies on radiomics in patients with LARC concentrated on whole tumor features in patients treated with nCRT. Palmisano and colleagues (25) analyzed the apparent diffusion coefficient (ADC) and imaging *via* DWI and T2WI before, during, and after nCRT in patients with LARC and demonstrated that changes in the ADC value and tumor volume at different times could help identify pCR. Shaish et al. (26) reported that T2WI radiomics could be used to predict pCR, neoadjuvant rectal scores, and TRG in patients with LARC receiving nCRT. Furthermore, Petresc et al. (27) reported that T2WI radiomics showed good predictive performance for LARC non-response. In our research, we proposed a pretreatment MRI-based peri- and intratumor features model combining clinical and radiomic features to predict a favorable response to NAC in patients with LARC. Interestingly, the model combining intratumor and clinical features did not show statistically significant improvement in the predictive performance compared to that of the radiomics model. The reason for this may be that the radiomics model yielded good performance in predicting treatment responses in patients with LARC, and the partial clinical characteristics were reflected in the radiomics. Although prediction performance varied in the validation cohort, the model incorporating peritumor features performed as well as that incorporating intratumor features in predicting good treatment response.

To our knowledge, this is the first study to evaluate the performance of a model including both peritumor and intratumor radiomics features in predicting a good response to NAC in LARC. Delli Pizzi et al. (28) reported that a model combining primary staging and radiomics, including information on tumor cores and borders, performed best in predicting the response to CRT. In that study, they included 72 patients with LARC who received nCRT for analysis, and those with TRGs of 1 or 2 were defined as good responders (this was the definition within our study as well). For image feature analysis, machine-learning approaches were adopted to establish the model. Models evaluating tumor core and tumor border radiomics and clinical features yielded AUC values of 0.689 and 0.541, respectively; the combined model improved the AUC to 0.793 (z=4.00, p=5.6×10^-5^). The tumor border model performed poorly in predicting treatment response. However, in our study, the model combining peri- and intratumor radiomics features did not improve the AUC significantly compared to the individual radiomics models. This may be because of differences between the treatment program and image processing. In contrast, a study by Hu et al. (20) found that the performance of the peritumor model was better than that of the intratumor model in predicting treatment response within the training set among patients with esophageal squamous cell carcinoma; the performance of the two models was similar in the test set as well. Our results showed that there were no significant differences in performance between peritumor and intratumor radiomics models, which was in agreement with the study conducted by Hu et al. (20). Li and colleagues (29) constructed a multimodal model to predict good treatment response in patients with LARC receiving NAC, including CT, HR-T2WI, DCE-T1WI, and ADC and demonstrated that the HR-T2WI model showed the best performance. However, in that study, the authors did not include peritumor information. Braman and colleagues (30) observed that a model combining intra- and peritumor radiomic features showed good performance in predicting pCR to NAC based on pretreatment breast DCE-MRI and suggested that the radiomic feature performance for predicting response was associated with breast tumor receptor subtypes. Furthermore, Wu and colleagues (22) identified that the enhancement patterns of the tumor-adjacent tissue obtained *via* DCE-MRI might be related to the signaling pathways involved in tumor necrosis as well as a worse prognosis in patients with breast cancer. Thus, peritumor imaging features contain overlooked information related to treatment response. Combining intra-peritumor radiomics features and clinical characteristics can thus be expected to improve prediction performance.

Peritumor characteristics were associated with treatment response, which may be related to the peritumor stroma, lymphocyte infiltration, and immune microenvironment. This suggests that the stroma in rectal cancer contains important information concerning the prognosis and treatment response. Various studies have also revealed that the response to chemo- or radiotherapy may be related to stoma cells or lymphocytes in other malignant neoplasms (31). Our results demonstrate that a peritumor radiomics model performs similarly to an intratumor radiomics model in predicting treatment response. This indicates that peritumor tissue might contain components that influence the treatment response and implies the importance of predictive information within peritumor radiomics features.

### Limitations

Our study proved that combining peri- and intratumor radiomics could predict a good response to NAC. However, our study has several limitations. First, because of the single-center design, small sample design study, selection bias is inevitable during patient recruitment. Besides, lack of standard protocol of acquisition of image also leads to selection bias. Second, Evaluating the peritumor area relies on experienced radiologists and requires substantial time and effort. The variability between the inter-reader may lead to poor reproducibility. Third, we only analyzed the impact of peritumor features on neoadjuvant treatment response at the theoretical level and have not further confirmed these findings at the molecular level. Thus, it is impossible to clarify the pathophysiological process underlying the impact of the tumor perimeter on neoadjuvant treatment response.

## Conclusions

Our study demonstrated that peritumor radiomics features contained important information related to a favorable response to NAC. We found that a model combining the clinical characteristics and intra-peritumor radiomics features could improve predictive capability in terms of identifying a good response to NAC in patients with LARC. Predictive models are commonly used in precision medicine. The results of our study inform future research directions and, if confirmed, will inform medical guidelines and optimal clinical decision-making in personalized medicine.

## Data availability statement

The original contributions presented in the study are included in the article/**Supplementary Material**. Further inquiries can be directed to the corresponding authors.

## Ethics Statement

The study design was approved by the ethics committee of the Sun Yat-sen University Cancer Center (Approval no. B2021-157-01).

## Author Contributions

B-YC designed the study. YL, X-HJ, LX, and X-FL organized the data. HJ and HX analyzed and visualized the data. B-YC drafted the article. X-FT, P-QC, and LL revised the paper. All authors contributed to the article and approved the submitted version.

## Funding

This work was supported by The Youth Fund Project of Guangdong Basic and Applied Basic Research Fund Regional Joint Fund (2020A1515110939).

## Conflict of Interest

The authors declare that the research was conducted in the absence of any commercial or financial relationships that could be construed as a potential conflict of interest.

## Publisher’s Note

All claims expressed in this article are solely those of the authors and do not necessarily represent those of their affiliated organizations, or those of the publisher, the editors and the reviewers. Any product that may be evaluated in this article, or claim that may be made by its manufacturer, is not guaranteed or endorsed by the publisher.

## Supplementary Material

Supplementary Figure 1 Texture feature selection using the least absolute shrinkage and selection operator binary logistic regression model. **(A, B)** Selection of peritumor radiomics. **(C, D)** Selection of intratumor radiomics.

## References

[1] Sung H Ferlay J Siegel RL Laversanne M Soerjomataram I Jemal A. Global Cancer Statistics 2020: GLOBOCAN Estimates of Incidence and Mortality Worldwide for 36 Cancers in 185 Countries. CA Cancer J Clin (2021) 71(3):209–49. doi: 10.3322/caac.21660 33538338

[2] Liu Z Zhang XY Shi YJ Wang L Zhu HT Tang Z. Radiomics Analysis for Evaluation of Pathological Complete Response to Neoadjuvant Chemoradiotherapy in Locally Advanced Rectal Cancer. Clin Cancer Res (2017) 23(23):7253–62. doi: 10.1158/1078-0432.CCR-17-1038 28939744

[3] Bahadoer RR Dijkstra EA van Etten B Marijnen CAM Putter H Kranenbarg EM. Short-Course Radiotherapy Followed by Chemotherapy Before Total Mesorectal Excision (TME) Versus Preoperative Chemoradiotherapy, TME, and Optional Adjuvant Chemotherapy in Locally Advanced Rectal Cancer (RAPIDO): A Randomised, Open-Label, Phase 3 Trial. Lancet Oncol (2021) 22(1):29–42. doi: 10.1016/S1470-2045(20)30555-6 33301740

[4] Giunta EF Bregni G Pretta A Deleporte A Liberale G Bali AM. Total Neoadjuvant Therapy for Rectal Cancer: Making Sense of the Results From the RAPIDO and PRODIGE 23 Trials. Cancer Treat Rev (2021) 96:102177. doi: 10.1016/j.ctrv.2021.102177 33798955

[5] Habr-Gama A Perez RO Nadalin W Sabbaga J Ribeiro U Jr Silva e Sousa AH Jr. Operative Versus Nonoperative Treatment for Stage 0 Distal Rectal Cancer Following Chemoradiation Therapy: Long-Term Results. Ann Surg (2004) 240(4):711–717; discussion 717-718. doi: 10.1097/01.sla.0000141194.27992.32 15383798 PMC1356472

[6] Deng Y Chi P Lan P Wang L Chen W Cui L. Modified FOLFOX6 With or Without Radiation Versus Fluorouracil and Leucovorin With Radiation in Neoadjuvant Treatment of Locally Advanced Rectal Cancer: Initial Results of the Chinese FOWARC Multicenter, Open-Label, Randomized Three-Arm Phase III Trial. J Clin Oncol (2016) 34(27):3300–7. doi: 10.1200/JCO.2016.66.6198 27480145

[7] Deng Y Chi P Lan P Wang L Chen W Cui L Neoadjuvant Modified FOLFOX6 With or Without Radiation Versus Fluorouracil Plus Radiation for Locally Advanced Rectal Cancer: Final Results of the Chinese FOWARC Trial. J Clin Oncol (2019) 37(34):3223–3. doi: 10.1200/JCO.18.02309. PMC6881102 31557064

[8] Ogura A Uehara K Aiba T Sando M Tanaka A Ohara N. Indications for Neoadjuvant Treatment Based on Risk Factors for Poor Prognosis Before and After Neoadjuvant Chemotherapy Alone in Patients With Locally Advanced Rectal Cancer. Eur J Surg Oncol (2021) 47(5):1005–11. doi: 10.1016/j.ejso.2020.10.038 33189492

[9] Torkzad MR Pahlman L Glimelius B. Magnetic Resonance Imaging (MRI) in Rectal Cancer: A Comprehensive Review. Insights Imaging (2010) 1(4):245–67. doi: 10.1007/s13244-010-0037-4 PMC3259411 22347920

[10] Caruso D Zerunian M De Santis D Biondi T Paolantonio P Rengo M. Magnetic Resonance of Rectal Cancer Response to Therapy: An Image Quality Comparison Between 3.0 and 1.5 Tesla. BioMed Res Int (2020) 2020:9842732. doi: 10.1155/2020/9842732 33102603 PMC7576357

[11] Caruso D Polici M Zerunian M Pucciarelli F Guido G Polidori T. Radiomics in Oncology, Part 1: Technical Principles and Gastrointestinal Application in CT and MRI. Cancers (Basel) (2021) 13(11):2522. doi: 10.3390/cancers13112522 34063937 PMC8196591

[12] Lambin P Rios-Velazquez E Leijenaar R Carvalho S van Stiphout RG Granton P. Radiomics: Extracting More Information From Medical Images Using Advanced Feature Analysis. Eur J Cancer (2012) 48(4):441–6. doi: 10.1016/j.ejca.2011.11.036 PMC4533986 22257792

[13] Coppola F Giannini V Gabelloni M Panic J Defeudis A Lo Monaco S. Radiomics and Magnetic Resonance Imaging of Rectal Cancer: From Engineering to Clinical Practice. Diagnostics (Basel) (2021) 11(5):756. doi: 10.3390/diagnostics11050756 33922483 PMC8146913

[14] Bulens P Couwenberg A Intven M Debucquoy A Vandecaveye V Van Cutsem E. Predicting the Tumor Response to Chemoradiotherapy for Rectal Cancer: Model Development and External Validation Using MRI Radiomics. Radiother Oncol (2020) 142:246–52. doi: 10.1016/j.radonc.2019.07.033 PMC6997038 31431368

[15] Chen H Shi L Nguyen KNB Monjazeb AM Matsukuma KE Loehfelm TW. MRI Radiomics for Prediction of Tumor Response and Downstaging in Rectal Cancer Patients After Preoperative Chemoradiation. Adv Radiat Oncol (2020) 5(6):1286–95. doi: 10.1016/j.adro.2020.04.016 PMC7718560 33305090

[16] Horvat N Veeraraghavan H Khan M Blazic I Zheng J Capanu M. MR Imaging of Rectal Cancer: Radiomics Analysis to Assess Treatment Response After Neoadjuvant Therapy. Radiology (2018) 287(3):833–43. doi: 10.1148/radiol.2018172300 PMC5978457 29514017

[17] Li Z Ma X Shen F Lu H Xia Y Lu J. Evaluating Treatment Response to Neoadjuvant Chemoradiotherapy in Rectal Cancer Using Various MRI-Based Radiomics Models. BMC Med Imaging (2021) 21(1):30. doi: 10.1186/s12880-021-00560-0 33593304 PMC7885409

[18] Wan L Peng W Zou S Ye F Geng Y Ouyang H. MRI-Based Delta-Radiomics are Predictive of Pathological Complete Response After Neoadjuvant Chemoradiotherapy in Locally Advanced Rectal Cancer. Acad Radiol (2021) 28(Suppl 1):S95–S104. doi: 10.1016/j.acra.2020.10.026 33189550

[19] Khorrami M Khunger M Zagouras A Patil P Thawani R Bera K. Combination of Peri- and Intratumoral Radiomic Features on Baseline CT Scans Predicts Response to Chemotherapy in Lung Adenocarcinoma. Radiol Artif Intell (2019) 1(2):e180012. doi: 10.1148/ryai.2019180012 32076657 PMC6515986

[20] Hu Y Xie C Yang H Ho JWK Wen J Han L. Assessment of Intratumoral and Peritumoral Computed Tomography Radiomics for Predicting Pathological Complete Response to Neoadjuvant Chemoradiation in Patients With Esophageal Squamous Cell Carcinoma. JAMA Netw Open (2020) 3(9):e2015927. doi: 10.1001/jamanetworkopen.2020.15927 32910196 PMC7489831

[21] Mandard AM Dalibard F Mandard JC Marnay J Henry-Amar M Petiot JF. Pathologic Assessment of Tumor Regression After Preoperative Chemoradiotherapy of Esophageal Carcinoma. Cancer (1994) 73(11):2680–6. doi: 10.1002/1097-0142(19940601)73:11<2680::aid-cncr2820731105>3.0.co;2-c 8194005

[22] Smith NJ Barbachano Y Norman AR Swift RI Abulafi AM Brown G. Prognostic Significance of Magnetic Resonance Imaging-Detected Extramural Vascular Invasion in Rectal Cancer. Br J Surg (2008) 95(2):229–36. doi: 10.1002/bjs.5917 17932879

[23] Detering R van Oostendorp SE Meyer VM van Dieren S Bos ACRK Dekker JWT. MRI Ct1-2 Rectal Cancer Staging Accuracy: A Population-Based Study. Br J Surg (2020) 107(10):1372–82. doi: 10.1002/bjs.11590 PMC7496930 32297326

[24] Arya S Sen S Engineer R Saklani A Pandey T. Imaging and Management of Rectal Cancer. Semin Ultrasound CT MR (2020) 41(2):183–206. doi: 10.1053/j.sult.2020.01.001 32446431

[25] Palmisano A Di Chiara A Esposito A Rancoita PMV Fiorino C Passoni P. MRI Prediction of Pathological Response in Locally Advanced Rectal Cancer: When Apparent Diffusion Coefficient Radiomics Meets Conventional Volumetry. Clin Radiol (2020) 75(10):798.e1–.e11. doi: 10.1016/j.crad.2020.06.023 32712007

[26] Shaish H Aukerman A Vanguri R Spinelli A Armenta Zhang P Jambawalikar S. Radiomics of MRI for Pretreatment Prediction of Pathologic Complete Response, Tumor Regression Grade, and Neoadjuvant Rectal Score in Patients With Locally Advanced Rectal Cancer Undergoing Neoadjuvant Chemoradiation: An International Multicenter Study. Eur Radiol (2020) 30(11):6263–73. doi: 10.1007/s00330-020-06968-6 32500192

[27] Petresc B Lebovici A Caraiani C Feier DS Graur F Buruian MM. Pre-Treatment T2-WI Based Radiomics Features for Prediction of Locally Advanced Rectal Cancer Non-Response to Neoadjuvant Chemoradiotherapy: A Preliminary Study. Cancers (Basel) (2020) 12(7):1984. doi: 10.3390/cancers12071894 PMC7409205 32674345

[28] Delli Pizzi A Chiarelli AM Chiacchiaretta P d'Annibale M Croce P Rosa C. MRI-Based Clinical-Radiomics Model Predicts Tumor Response Before Treatment in Locally Advanced Rectal Cancer. Sci Rep (2021) 11(1):5379. doi: 10.1038/s41598-021-84816-3 33686147 PMC7940398

[29] Li ZY Wang XD Li M Liu XJ Ye Z Song B. Multi-Modal Radiomics Model to Predict Treatment Response to Neoadjuvant Chemotherapy for Locally Advanced Rectal Cancer. World J Gastroenterol (2020) 26(19):2388–402. doi: 10.3748/wjg.v26.i19.2388 PMC7243642 32476800

[30] Braman NM Etesami M Prasanna P Dubchuk C Gilmore H Tiwari P. Intratumoral and Peritumoral Radiomics for the Pretreatment Prediction of Pathological Complete Response to Neoadjuvant Chemotherapy Based on Breast DCE-MRI. Breast Cancer Res (2017) 19(1):57. doi: 10.1186/s13058-017-0846-1 28521821 PMC5437672

[31] Post AEM Smid M Nagelkerke A Martens JWM Bussink J Sweep FCGJ. Interferon-Stimulated Genes Are Involved in Cross-Resistance to Radiotherapy in Tamoxifen-Resistant Breast Cancer. Clin Cancer Res (2018) 24(14):3397–408. doi: 10.1158/1078-0432.CCR-17-2551 29661777

