# Supplementary figures and images for: MRI-Based Radiomics Features to Predict Treatment Response to Neoadjuvant Chemotherapy in Locally Advanced Rectal Cancer: A Single Center, Prospective Study
Source: Front Oncol. 2022 May 12;12:801743. doi: 10.3389/fonc.2022.801743 (PMC9133669; doi:10.3389/fonc.2022.801743)

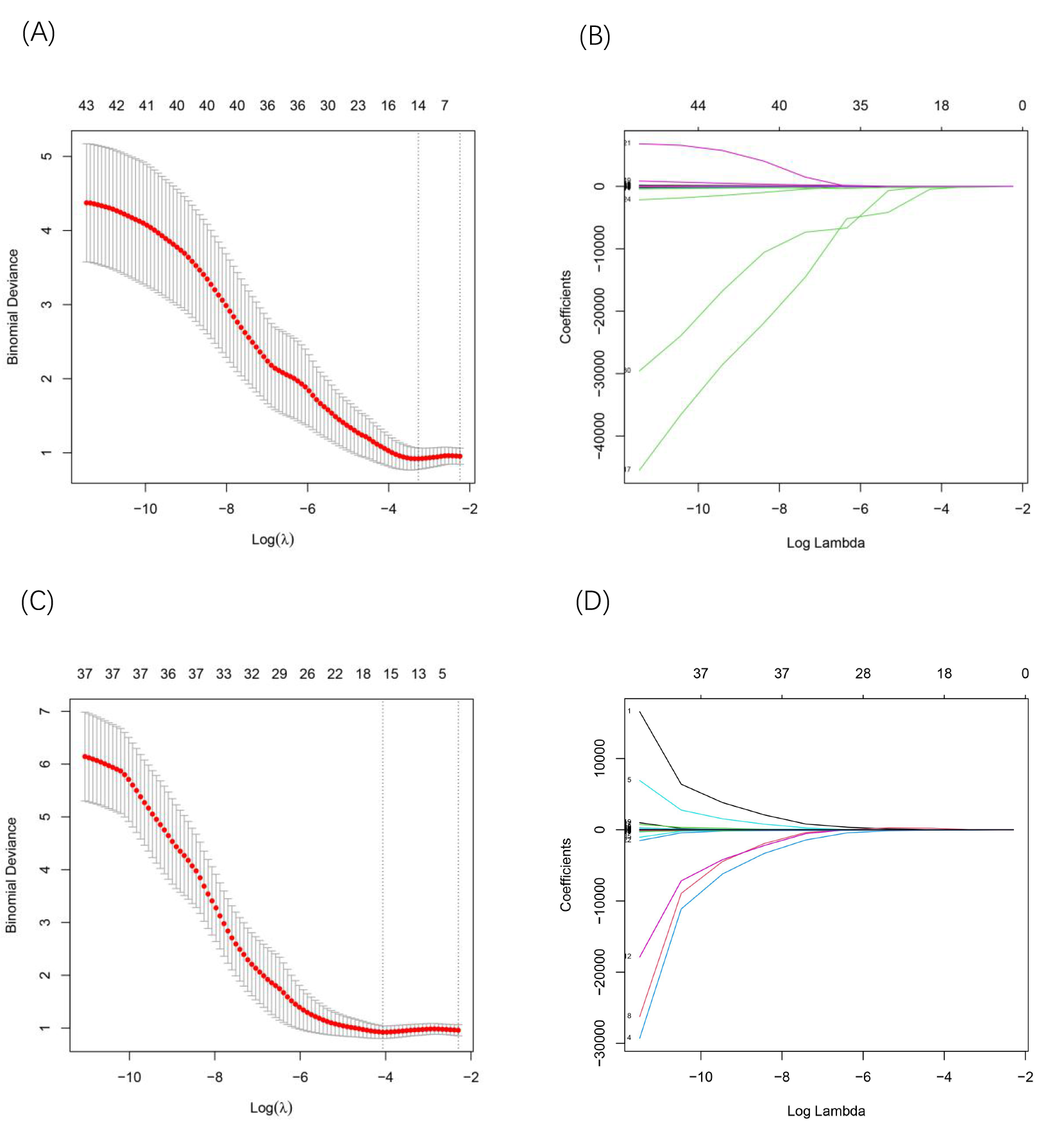

Supplement: Supplementary Figure 1 — Texture feature selection using the least absolute shrinkage and selection operator binary logistic regression model. (A, B) Selection of peritumor radiomics. (C, D) Selection of intratumor radiomics. [file Image_1.tif]
