# Supplementary material for: MRI-Based Radiomics Features to Predict Treatment Response to Neoadjuvant Chemotherapy in Locally Advanced Rectal Cancer: A Single Center, Prospective Study
Source: Front Oncol. 2022 May 12;12:801743. doi: 10.3389/fonc.2022.801743 (PMC9133669; doi:10.3389/fonc.2022.801743)
Supplement: Supplementary file 2 [file DataSheet_1.pdf]

Supplement. Parameter settings for image and radiomics feature extraction.

```
import nrrd
import numpy as np
from scipy.ndimage import zoom, binary_dilation
import os
import cv2
import pandas as pd
import SimpleITK as sitk
from radiomics import featureextractor
import six
import sys
def read_nrrd(dataDir):
    data, options = nrrd.read(dataDir)
    return data, options
def resampling(data, old_spacing, new_spacing, order=2):
    new_shape = np.round(data.shape * old_spacing / new_spacing)
    resize_factor = new_shape / data.shape
    new_data = zoom(data, resize_factor, mode='nearest', order=order)
    return new_data
def dilation(data, radius):
    rad = 2 * radius + 1
    data = data.astype(np.uint8)
    dil_data = binary_dilation(data, structure=np.ones((rad, rad, rad))).astype(np.uint8)
    return dil_data
def intersection(data1, data2):
    return (data1 * data2).astype(np.uint8)
def union(data1, data2):
    return ((data1 + data2) > 0.5).astype(np.uint8)
def check_dir(dir):
    if not os.path.exists(dir):
        os.makedirs(dir)
def save_img(data, dir, type):
    rootdir = os.path.join(dir, type)
    check_dir(rootdir)
    data = (data * 255).astype(np.uint8)
    for i in range(data.shape[-1]):
        img = data[:, :, i]
        cv2.imwrite(os.path.join(rootdir, str(i)+'.jpg'), img)
def directed_dilation(dataDir):
    img, imgop = read_nrrd(dataDir)
    mask, maskop = read_nrrd(dataDir[:-5]+'-.nrrd')
    x_spacing = float(imgop['0028|0030'].split('\\\\')[0])
    y_spacing = float(imgop['0028|0030'].split('\\\\')[1])
```

```
        z_spacing = float(imgop['0018|0088'])
        old_spacing = np.array([x_spacing, y_spacing, z_spacing])
        new_spacing = np.array([1, 1, 1])
        lab1 = (mask == 1).astype(np.uint8)
        lab2 = (mask == 2).astype(np.uint8)
        imgrsp = resampling(img, old_spacing, new_spacing)
        lab1rsp = resampling(lab1, old_spacing, new_spacing)
        # save_img(lab1rsp, testdir, 'lab1rsp')
        lab2rsp = resampling(lab2, old_spacing, new_spacing)
        # save_img(lab2rsp, testdir, 'lab2rsp')
        lab1rspdil1 = dilation(lab1rsp, radius=1)
        # save_img(lab1rspdil1, testdir, 'lab1rspdil1')
        lab1rspdil1nlab2rsp = intersection(lab1rspdil1, lab2rsp)
        # save_img(lab1rspdil1nlab2rsp, testdir, 'lab1rspdil1nlab2rsp')
        lab1rspdil1nlab2rspdil5 = dilation(lab1rspdil1nlab2rsp, radius=5)
        # save_img(lab1rspdil1nlab2rspdil5, testdir, 'lab1rspdil1nlab2rspdil5')
        lab1rspdil1nlab2rspdil5ulab1rsp = union(lab1rspdil1nlab2rspdil5, lab1rsp)
        # save_img(lab1rspdil1nlab2rspdil5ulab1rsp, testdir,
'lab1rspdil1nlab2rspdil5ulab1rsp')
        return imgrsp, lab1rspdil1nlab2rspdil5ulab1rsp
def source_data(dataDir, order = 1):
        # order: 0-source; 1-mask1; 2-mask2; 3-source-mask1; 4-source-mask1-mask2
        img, imgop = read_nrrd(dataDir)
        mask, maskop = read_nrrd(dataDir[:-5] + '-.nrrd')
        x_spacing = float(imgop['0028|0030'].split('\\\\')[0])
        y_spacing = float(imgop['0028|0030'].split('\\\\')[1])
        z_spacing = float(imgop['0018|0088'])
        old_spacing = np.array([x_spacing, y_spacing, z_spacing])
        new_spacing = np.array([1, 1, 1])
        lab1 = (mask == 1).astype(np.uint8)
        lab2 = (mask == 2).astype(np.uint8)
        if order == 0:
                return resampling(img, old_spacing, new_spacing)
        elif order == 1:
                return resampling(lab1, old_spacing, new_spacing)
        elif order == 2:
                return resampling(lab2, old_spacing, new_spacing)
        elif order == 3:
                return resampling(img, old_spacing, new_spacing), resampling(lab1,
old_spacing, new_spacing)
        elif order == 4:
                return resampling(img, old_spacing, new_spacing), resampling(lab1,
old_spacing, new_spacing), resampling(lab2, old_spacing, new_spacing)
        else:
```

```
            print('Error order value')
            sys.exit(0)
def extract_radiomics(datadir, outputdir, order=0):
    check_dir(outputdir)
    extractor = featureextractor.RadiomicsFeatureExtractor()
    extractor.enableAllImageTypes()
    extractor.enableAllFeatures()
    dict = {}
    for file in os.listdir(datadir):
        if '-.nrrd' in file:
            continue
        patient = file[:-5]
        # Choose data type: directed_dilation or source_data
        if order == 1:
            img, mask = directed_dilation(os.path.join(datadir, file))
        elif order == 0:
            img, mask = source_data(os.path.join(datadir, file), order=3)
        else:
            print('Error order value')
            sys.exit(0)
        sitk_img = sitk.GetImageFromArray(img)
        sitk_img.SetSpacing((1, 1, 1))
        sitk_mask = sitk.GetImageFromArray(mask)
        sitk_mask.SetSpacing((1, 1, 1))
        result = extractor.execute(sitk_img, sitk_mask)
        if 'patient' in dict:
            dict['patient'].append(patient)
        else:
            dict['patient'] = [patient]
        cnt = 0
        for key, value in six.iteritems(result):
            cnt += 1
            if cnt <= 37:
                continue
            if key in dict:
                dict[key].append(value)
            else:
                dict[key] = [value]
    dataframe = pd.DataFrame(dict)
    if order == 1:
        dataframe.to_csv(os.path.join(outputdir,             'feature_directed_dil.csv'),
index=False, sep=',')
    else:
        dataframe.to_csv(os.path.join(outputdir,             'feature_source_data.csv'),
```

```
index=False, sep=',')
def calc_volume(datadir, outputdir):
    check_dir(outputdir)
    dict = {}
    dict['patient'] = []
    dict['cubic millimeter'] = []
    for file in os.listdir(datadir):
        if '-.nrrd' in file:
            continue
        patient = file[:-5]
        mask = source_data(os.path.join(datadir, file), order=1)
        vol = np.sum(mask)
        dict['patient'].append(patient)
        dict['cubic millimeter'].append(vol)
    dataframe = pd.DataFrame(dict)
    dataframe.to_csv(os.path.join(outputdir, 'volume_source_data.csv'), index=False,
sep=',')
if __name__ == '__main__':
    dataDir = r'path/to/source data'
    dstDir = r'path/to/output dir'
    extract_radiomics(dataDir, dstDir)
```
