# Supplementary material for: MRI-Based Radiomics Features to Predict Treatment Response to Neoadjuvant Chemotherapy in Locally Advanced Rectal Cancer: A Single Center, Prospective Study
Source: Front Oncol. 2022 May 12;12:801743. doi: 10.3389/fonc.2022.801743 (PMC9133669; doi:10.3389/fonc.2022.801743)
Supplement: Supplementary file 3 [file DataSheet_2.pdf]

Supplemental table 1. Features that remained after each selection step for radiomics signature construction.

features	
directed_data	source_data
exponential_firstorder_Kurtosis	gradient_glcm_Correlation
logarithm_glcm_Idmn	logarithm_gldm_SmallDependenceLowGrayLevelEmphasis
square_glszm_GrayLevelNonUniformity	logarithm_glrlm_LongRunHighGrayLevelEmphasis
square_ngtdm_Strength	wavelet.LLH_glcm_Imc1
wavelet.LLH_glcm_Correlation	wavelet.LHL_firstorder_Kurtosis
wavelet.LHH_firstorder_Mean	wavelet.LHH_firstorder_Skewness
wavelet.HLH_firstorder_Mean	wavelet.LHH_glszm_ZoneEntropy
wavelet.HLH_firstorder_Median	wavelet.HHL_firstorder_Mean
wavelet.HHH_firstorder_Median	wavelet.HHH_firstorder_Median
	wavelet.HHH_gldm_DependenceVariance
